# Prevalence and factors associated with intestinal parasitic infections among food handlers of Southern Ethiopia: cross sectional study

**DOI:** 10.1186/s12889-016-2790-x

**Published:** 2016-02-01

**Authors:** Mohammedaman Mama, Getaneh Alemu

**Affiliations:** Department of Medical Laboratory Science, College of Medicine and Health Sciences, Arba Minch University, Arba Minch, Ethiopia

**Keywords:** Intestinal parasites, Food handlers, Ethiopia

## Abstract

**Background:**

Globally about one third of the total population is estimated to be infected with intestinal parasites, of which, the majority are people living in tropical and sub-tropical parts of the world. Cases of intestinal parasitosis are also highly abundant in Ethiopia and hence the aim of present study was to assess prevalence and predictors of intestinal parasitic infections among food handlers working in Arba Minch University students’ cafeteria, South Ethiopia.

**Method:**

A cross sectional study was conducted among food handlers working in Arba Minch University from April to June, 2015. A pretested structured questionnaire was used for collecting data about socio-demographic characteristics and possible risk factors. Stool specimens were collected and examined microscopically for the presence of eggs, cysts and trophozoites of intestinal parasites. Data entry and analysis were done using SPSS version 20 software.

**Results:**

A total of 376 food handlers were enrolled in the study of which thirty one of them were not willing to participate for a stool examination. The majority of study participants were females 273 (72.6 %). About 123 (36 %) of food handlers were found to be positive for different intestinal parasites with the most abundant parasite of Entamoeba histolytica/dispar 48 (14 %) followed by Ascaris lumbricoides 32 (9.27 %). Finger nail status (AOR: 2.2, 95 % CI: 1.29–3.72), hand washing practice after toilet (AOR: 1.71, 95 % CI: 1.06–2.77), hand washing practice before food handling (AOR: 1.69, 95 % CI: 1.04–2.75), preparing food when suffering from diseases (AOR: 3.08, 95 % CI: 1.17–8.13), and using common knife for cutting raw flesh food and other food (AOR: 1.72, 95 % CI: 1.01–2.92) were independent predictors of intestinal parasitic infection among the food handlers.

**Conclusion:**

This study revealed a high prevalence of intestinal parasites among food handlers. Since most of the intestinal parasites are transmitted by the feco-oral route, food handlers could be an important source of infection to the students and general population. Therefore, constant epidemiological surveillance through biannual routine parasitological tests and treatment of the infected cases along with the improvement of personal hygiene and environmental sanitation are recommended to control the parasitic infection in food handlers.

## Background

Food borne diseases are public health problems worldwide. It was estimated that about 30 % of the population living in the developed world suffers from diarrheal diseases, mostly caused by food borne microbial pathogens. About 2 million deaths occur annually due to food borne diseases in developing countries [1, 2].

Globally about one third of the total population is estimated to be infected with intestinal parasites, the majority being people living in tropical and sub-tropical parts of the world [3]. About 819 million people are infected with *Ascaris lumbricoides* (*A. lumbricoides*), 464.6 million people with *Trichuris trichiura* (*T. trichuira*), 438.9 million people with hookworm infection [4], 500 million people with *Entamoeba histolytica (E. histolytica)*, and 2.8 million people are infected with *Giardia lamblia* (*G. lamblia*) [5].

As in many developing countries, cases of intestinal parasitosis are also highly abundant in Ethiopia. It is estimated that one third of Ethiopians are infected with *A. lumbricoides*, one quarter is infected with *T. trichiura* and one in eight lives with hookworm. As a result, Ethiopia has the second highest burden of ascariasis, the third highest burden of hookworm, and the fourth highest burden of trichuriasis in Sub-Saharan Africa [6].

As a result of this, the Federal Ministry of Health (FMoH) of Ethiopia has prioritized intestinal parasitic infection as one of the Neglected Tropical Diseases (NTDs) in the National Master Plan of NTDs, to address the public health problems due to NTDs [7].

High prevalence of intestinal parasitic infections and poly parasitism affect the health status of individuals mainly affecting physical and mental developments causing malnutrition, anaemia, stunting, cognitive impairment, lowered educational achievement and interfering with productivity [8, 9]. It was estimated and indicated by different researchers; high prevalence of intestinal parasites is largely due to lack of personal and environmental sanitation, lack of safe water supply, human behavior, poverty, ignorance of health promotion practices and impoverished health services [10–12].

Transmission of intestinal parasites is effected directly or indirectly through faeces contaminated objects such as food, water, soil and finger. Although various modes of transmission of intestinal parasites are known to exist, several studies have shown the higher magnitude of hand to mouth transmission as potential sources of exposure to parasitic infections [13, 14]. Accordingly, food handlers with poor personal hygiene working in food serving establishments could be potential sources of infections by many of the intestinal helminths and protozoa. Therefore, food handlers with poor personal hygiene working in food service establishments could be potential sources of infections [15, 16].

Various studies have been conducted to assess how prevalent intestinal parasitic infections among food handlers in different parts of the world including Ethiopia. In Ethiopia, the rate of infection with intestinal parasites among food handlers ranged from 29 to 72 % with different reports on the predominant species of parasites and hygiene practices [1, 10, 13, 14]. The importance of food handlers as threats in the transmissions of parasitic diseases has been stressed by several authors [9, 17, 18] and therefore, the aim of this study was to determine intestinal prevalence and associated risk factors among food handlers working in Arba Minch University Students’ Cafeteria.

## Methods

### Study area, design and period

A cross sectional study design was conducted at Arba Minch Universityfrom April- June, 2015. Arba Minch University is one of the well-established universities found in the Southern Nations, Nationalities and People's Region (SNNPR). It is located at Arba Minch town, 500 km south of Addis Ababa. The main campus of the university is situated at the eastern foot of Gamo mountain ranges and adjacent to the vast low land stretching towards Lake Abaya and Lake Chamo which form part of the East African Rift Valley. The spectacular features of the twin Rift Valley lakes, Abaya and Chamo, impart a picturesque view to the University as it is viewed from the main campus hills. Its two new campuses are named after these two lakes while the third new campus is named after one of the wonderful national parks of the country – Nech Sar. The gifted land of the South offers a huge opportunity to the University to venture into new territories of education, research and development. At present, the University runs both undergraduate and post graduate programs in five campuses with 17,932 total number of students which enrolled in the regular and continuing education programs.

### Sample size determination and sampling technique

The sample size was determined using sample size determination for estimation of single population proportion formula. Taking 63 % parasite prevalence from previous study [19], 95 % confidence interval (z = 1.96) and 5 % marginal error (d = 0.05) the initial sample size was 358 and, finally by considering a 5 % (≈18 subjects) non response rate, the final sample size was determined to be 376.

To select representative groups from 1000 number of total food handlers a proportional sample size was determined for each stratum (campuses), and food handlers were selected randomly by lottery method from the roster lists of food handlers which was obtained from cafeteria office of each Arba Minch University campuses.

### Data collection and laboratory processing

Data related to socio-demographic characteristics, and personal hygiene practices of food handlers and related risk factors was collected by face to face interview using pre tested structured questionnaire and observational guidelines. All the questionnaires were checked for accuracy and completeness. After proper instruction, food handlers were given labeled collection cups that contain applicator sticks. From each study subject, about 2g of fresh stool was collected. Each of the specimens was checked for its label, quantity and procedure of collection. A portion each of the stool samples was processed with a direct microscopic technique to detect cysts, trophozoites, eggs and larva of intestinal parasites immediately. The remaining part of the samples was transported to research laboratory. Stool examinations were performed using the formol-ether concentration technique. Both the 10× and 40× objectives were used for detection of eggs and larvae of helminth and cysts and trophozoites of protozoan parasites. Iodine solution was used to detect and identify cysts of protozoan parasites.

Well experienced and trained laboratory technologist was recruited for laboratory examination. Before the actual stool specimen’s examination for the study subjects, pre-test was conducted on stool samples collected from patients attending student clinic to look the reliability/reproducibility of stool examination procedure by the laboratory technologist with two different microscopes.

### Data analysis

Data was edited, cleaned, entered and analyzed using statistical package for social science (SPSS) version 20. Descriptive analysis such as frequencies and mean was used. Initially the association between each exposure and the presence of infection was assessed using the chi-square test, and odds ratio was computed to measure the strength of the association. Univariate and bivariate analysis were conducted and crude and adjusted odds ratio with 95 % CI were calculated for statistical significance tests. Variables with significant at *P* < 0.3 in a bivariate analysis were considered for multivariate analysis through multiple logistic regression model to look their relative effect on the outcome variable by controlling other possible confounding factors. *P*-value of ≤ 0.05 was considered to indicate statistical association. Finally the result was presented using tables.

### Ethical considerations

The study protocol was ethically approved by review boards of Arba Minch University College of Medicine and Health Sciences with project code of GOV/AMU/TH.5-2/CMHS/MLS/02/07. Letter of cooperation was written to each leader of the cafeteria. Informed verbal consent was obtained from each study participant. Strict confidentiality was maintained during the interview process as well as anonymity was kept during data processing and report writing. Food handlers positive for intestinal parasites were treated.

## Results

### Socio demographic characteristics

A total of 376 food handlers were selected and all of them agreed to participate so that the response rate was 100 %. About 7.4 % were aged less than 20 years and the majority (63.3 %) lay in the working age group of 21–35 years. Majority of the workers were females (72.6 %) and were educated up to primary level (36.4 %). Only 36.2 % food handlers had certificate in food training and none of them had medical check-up (Table 1).

**Table 1:** The prevalence of intestinal parasitic infection with respect to socio-demographic characteristics of food handlers at Arba Minch University students’ cafeteria, Arba Minch, South Ethiopia, April- June 2015

Demographic characters	Infected	Not infected	*X* ^2^	*p*-value
	No. (%)	No. (%)		
Sex				
Male	41 (40)	62 (60)	4.36	0.037
Female	78 (28.6)	195 (71.4)		
Age in years				
≤ 20	14 (50)	14 (50)	8.05	0.045
21–35	71 (29.8)	167 (70.2)		
36–50	34 (33)	69 (67)		
≥ 50	0	7 (100)		
Years of service				
< 1 year	15 (58)	11 (42)	11.12	0.011
1–5 years	49 (30)	112 (70)		
6–10years	38 (32)	79 (68)		
> 10 years	17 (23.6)	55 (76.4)		
Educational status				
Illiterate	11 (31)	24 (69)	2.62	0.45
Primary school	40 (29.2	97 (70.8)		
Secondary school	29 (28.7)	72 (71.3)		
Higher than secondary school	39 (38)	64 (62)		
Certified in food training				
No	73 (30.4)	167 (69.6)	0.53	0.77
Yes	46 (33.8)	90 (66.2)		

### Prevalence of intestinal parasites

From the total study subjects (*n* = 376), thirty one of them were not willing to participate for a stool examination. As a result, a total of 345 food handlers participated for stool examination of whom, 123 (36 %) were found to be positive for various types of intestinal parasites. Of those infected, 66 (19.13 %) had helminthic infections, while the remaining 57 (16.52 %) had protozoan infection. The most abundant parasite was *E. histolytica/dispar* 48 (14 %) followed by *A. lumbricoides* 32 (9.3 %). Among 123 positive food handler’s, 2(0.6 %) had mixed infections (Table 2).

**Table 2:** Type and prevalence of intestinal parasites isolated from stool specimens of food handlers at Arba Minch University students’ cafeteria, Arba Minch, South Ethiopia, April- June 2015

Parasite Species	Frequency (%)
Protozoa	66 (19.13)
*E. histolytica/dispar*	48 (14)
Trophozoite form	35 (10.14)
Cyst form	13 (3.76)
*G. lamblia*	18 (5.22)
Trophozoite form	12 (3.5)
Cyst form	5 (1.44)
Helminthes	57 (16.52)
*A. lumbricoides*	31 (9)
*Taenia* species	14 (4.1)
*T. trichiura*	4 (1.2)
Hookworms	3 (0.9)
*S. stercoralis*	2 (0.6)
Mixed infection	2 (0.6)
Cyst of *E. histolytica/dispar* and *Teania* species	1 (0.3)
Ova of A. lumbricoides, *T. trichiura and* Hookworms	1 (0.3)

### Intestinal parasitic infections and associated factors

As shown in Table 3, different factors were assessed for possible association with intestinal parasitic infection among the study participants. The results of the study showed that 40 % (41/103) of male and 28.6 % (78/273) female participants were found to be infected with at least one parasite. The prevalence of infection with intestinal parasites have initial association with sex of the respondents (*p* = 0.038), However the association was not significant after adjusting for confounders using multivariate logistic regression (*p* = 0.530).

**Table 3:** Multivariate analysis of intestinal parasitic infections and potential risk factors among food handlers at Arba Minch University Students Cafeteria, Arba Minch, South Ethiopia, April- June 2015

Variables	Negative	Positive	Crude OR (95 % CI)	*P*-value	Adjusted OR (95 % CI)	*P*-value
	No. (%)	No. (%)				
Sex						
Male	62(60)	41(40)	1.653(1.029–2.656)	0.038	1.199(0.680–2.114)	0.530
Female	195(71.4)	78(28.6)	1.00		1.00	
Age						
< 20 years	14(50)	14(50)	2.352(1.066–5.188),	0.034	1.355(0.523–3.509)	0.531
21–35 years	167(70.2)	71(29.8)	1.00		1.00	
36–50 years	69(67)	34(33)	1.159(0.706–1.903)	0.560	1.162(0.664–2.033)	0.599
> 50 years	7(100)	0	0.000	0.999	0.000	0.999
Years of Service						
< 1 year	11(42)	15(58)	3.117 (1.336–7.273)	0.009	2.320 (0.887–6.068)	0.086
1–5 years	112(70)	49(30)	1.00		1.00	
6–10 years	79(68)	38(32)	1.099(0.659–1.835)	0.717	1.298 (0.724–2.329)	0.381
> 10 years	55(76.4)	17(23.6)	0.706(0.373–1.339)	0.287	.739 (0.351–1.557)	0.426
Finger nail Status						
Not trimmed	46(51)	44(49)	2.691(1.648–4.393)	0.000	2.193 (1.293–3.719)	0.004
Trimmed	211(74)	75(26)	1.00		1.00	

Food handlers aged below 20 years of age were found to have a high percentage (50 %) of infection as compared to other age groups. The association between age groups and intestinal parasitic infection was statistically significant (*p* = 0.034). After adjusting for possible confounders by multivariate logistic regression, the prevalence of infection with intestinal parasites was not significantly different among different age groups of food handlers. Similarly years of service does not have significant association with parasitic infection (AOR = 0.086). Forty four (49 %) of food handlers with untrimmed finger nails had infection with at least one parasite (Table 3). The finger nail status of the study participants had a significant association with the rate of intestinal parasitic infection (*p* = 0.004). The odds of parasitic infection was 2 times higher (AOR: 2.193, 95 % CI [1.29–3.72]) for food handlers who had untrimmed finger nail as compared to those who trimmed.

The practice of hand washing after toilet (*p* = 0.029) and before food handling (*p* = 0.034) was significantly associated with parasitic infection among the study participants. Food handler’s who were using water only when they washed their hands after toilet had a more likely risk of infection (with 71 %) for intestinal parasites [OR: 1.71, 95 % CI; (1.057–2.765)] than food handler’s who use water and soap. The extent of intestinal parasitosis was more likely to occur (with 69 %) among food handlers who washed their hands before food handling with water only [OR: 1.69, 95 % CI (1.04–2.75)] than food handlers who wash their hand with water and soap (Table 4).

**Table 4:** Multivariate analysis of intestinal parasitic infections and potential hygiene risk factors among food handlers at Arba Minch University Students Cafeteria, Arba Minch, South Ethiopia, April- June 2015

Variables	Negative	Positive	Crude OR (95 % CI)	*P*-value	Adjusted OR (95 % CI)	*P*-value
	No. (%)	No. (%)				
Hand washing after toilet						
With water only	100(61)	64(39)	1.827(1.178–2.834)	0.007	1.710 (1.057–2.765)	0.029
With water and soap	157(74)	55(26)	1.00		1.00	
Hand washing before food handling						
With water only	125(63)	74(27)	1.790(1.142–2.804)	0.011	1.691 (1.040–2.749)	0.034
With water and soap	130(75)	43(25)	1.00		1.00	
Preparing food when suffering from diseases like diarrhea, cold or skin diseases						
No	33(85)	6(15)	1.00		1.00	
Yes	224(66.5)	113(33.5)	0.360(0.147–0.885)	0.026	3.077(1.165–8.127)	0.023
Using common knife for cutting raw flesh food and other food						
No	97(74)	29(26)	1.00		1.00	
Yes	160(64)	90(36)	0.532(0.326–0.866)	0.011	1.715 (1.008–2.917)	0.046

Preparing food when suffering from disease like diarrhea, skin disease had a significant association with the rate of intestinal parasitic infection (*p* = 0.023). The odds of parasitic infection was 3 times higher (AOR: 3.077, 95 % CI [1.165–8.127]) for individuals who had disease and participate on food preparation as compared to those who did not prepare food when they are diseased. The practice of using common knife for cutting raw flesh food and other food had a statistically significant association with intestinal parasitic infection (*p* = 0.046) (Table 4). The multivariate logistic regression model estimated that individuals who use common knife for cutting raw flesh food and other food were 72 % times (AOR: 1.72, 95 % CI: 1.01–2.92) more likely to be infected with intestinal parasites than those who did not use common knife for cutting raw flesh food and other food.

## Discussion

Food handlers may be carrying a wide range of intestinal parasites and have been implicated in the transmission of many infections to the public in the community and to students in University. The spread of disease via food handlers is a common and persistent problem worldwide [13, 20]. Therefore, this study was undertaken to assess prevalence of intestinal parasites, among food handlers of Arba Minch University Student’s Cafeteria, Arba Minch, South Ethiopia.

In this study, most of food handlers working in students’ cafeteria of Arba Minch University were females, young adults and had low educational levels; which is in line with studies from different parts of Ethiopia like Bahir Dar, Gondar town and Addis Ababa [13, 14, 21]. The overall proportion of infected female food handlers (22.6 %) with intestinal parasites was higher than the proportion of infected male food handlers (12 %). This can be due to the fact that women are much more involved in kitchen work than men. Most of the males participate in the delivery of the already prepared food, while women are those who go bare footed during the preparation of the food, as well as those who do the washing of vegetables and fruits mainly in the kitchen.

Concerning the relation of age group and parasitic infection, the study revealed relatively a higher infection rate in the age group younger than 20 years. No significant difference was found in the distribution of parasitic infection among all age groups which shows that there is equal exposure to the infection and suggests an effect of environmental conditions on infection.

It is expected that all food handlers at University, military, hospitals etc. cafeterias to have a periodic medical checkup for food borne pathogens. Despite this fact, the interview result of our study showed that none of food handlers had a medical checkup for intestinal infection which agrees with study done in Bahir Dar town, Ethiopia [14].

In this study about 36 % of the food handlers were carriers of one or more of the pathogenic intestinal parasites. This is comparable with the finding of 38.1 % done in Nigeria [22], 32 % in Makka, Saudi Arabia [23] and 41.1 % in Bahir dar, Northwest Ethiopia [14]. However, it is higher than study of 1.3 to 7 % in India [16], 6.9 % in Omdurman, Sudan [24], 8.8 % in Turkey [25], 15.5 % in Sari, Northern Iran [11], 24.3 % in Gaza Strip, Palestine [26] and 29.1 % in Gondar, Northwest Ethiopia [21]. It was lower than the prevalence of 44.1 % from Jimma [1], 45.3 % from Addis Ababa [13], 49.4 % from Mekelle [10], 58.4 % from Jimma [9] and 71.8 % from Addis Ababa, Ethiopia [27]. The differences in reported prevalence in various studies may be due to socioeconomic status, climatic conditions, poverty, and personal and community hygiene.

In the present study, multiple intestinal parasitic infections were found with *A. lumbricoides* being the predominant parasite from helminthes followed by *Taenia* species, and *E. histolytica/dispar* was the most predominant from protozoan parasites followed by *G. lamblia*. High prevalence of ascariasis is a good indicator of improper fecal disposal, while that of entameobiasis reflects use of poor water quality among the study participants. This was consistent with the finding of a similar study conducted in Enugu State, Nigeria [28], and in different parts of Ethiopia [9, 10, 13, 14].

In this study encouraging results were obtained regarding practices of hand washing after toilet and before food preparation. Food handlers’ hand washing practices after toilet (100 %) was in parallel with the previous studies done in Ethiopia like, 90.6 % in Bahir dar [14]. However, hand washing practices of food handlers was low especially after touching body parts and after blowing nose which increase the likely hood of microorganisms cross contamination. These reflected that food handlers lack awareness about food contamination with poor hygienic practices.

Poor personal hygiene, including inadequate hand washing among food handlers is a common practice that contributes to food born diseases [10]. Parasite eggs in the soil can contaminate vegetables, then hands and hence directly enter into the mouth, or ingested by eating raw vegetables [11, 29].

This study identified high protozoan and helminthic infection that can easily be transmitted via feco-oral route, either directly from person to person or indirectly by eating or drinking fecally contaminated food and water. Hence, in this study multivariate logistic regression model indicated that untrimmed finger nail, hand washing practice after toilet, hand washing before food handling, preparing food when suffering from diseases like diarrhea and using common knife for cutting raw flesh food and other food were identified as determinant factors for food handlers being infected by intestinal parasites. The present study was subjected to the following limitations. The study was non-blinded. Due to lack of antigen tests, *Entamoeba histolytica* and *Entamoeba dispar* were not separated. This study did not attempt to assess the parasite carriage of the finger nail contents and parasite intensity due to logistic reasons. Specific methods such as the adhesive scotch tape for *E. vermicularis*, Harada Moori’s filter Paper for *S. stercoralis* and for hookworm infections was not done.

## Conclusion

This study revealed a high prevalence of intestinal parasites among food handlers. Since most of the intestinal parasites are transmitted by the feco-oral route, food handlers could be an important source of infection to the general population. Therefore, constant epidemiological surveillance through biannual routine parasitological tests and treatment of the infected cases along with the improvement of personal hygiene and environmental sanitation are recommended to control the parasitic infection in food handlers.
